# Medical Use of Polycatecholamines + Oxidoreductases-Modified Curdlan Hydrogels—Perspectives

**DOI:** 10.3390/ijms231710084

**Published:** 2022-09-03

**Authors:** Anna Michalicha, Agata Przekora, Dawid Stefaniuk, Magdalena Jaszek, Anna Matuszewska, Anna Belcarz

**Affiliations:** 1 Chair and Department of Biochemistry and Biotechnology, Medical University of Lublin, Chodzki 1, 20-093 Lublin, Poland; 2 Independent Unit of Tissue Engineering and Regenerative Medicine, Chair of Biomedical Sciences, Medical University of Lublin, Chodzki 1, 20-093 Lublin, Poland; 3 Department of Biochemistry and Biotechnology, Institute of Biological Sciences, Maria Curie-Skłodowska University, 20-031 Lublin, Poland

**Keywords:** polydopamine, poly(L-DOPA), immobilization, immunomodulation, compression, oxidoreductases

## Abstract

Curdlan (β-1,3-glucan), as a biodegradable polymer, is still an underestimated but potentially attractive matrix for the production of dressing materials. However, due to its lack of susceptibility to functionalization, its use is limited. The proposed curdlan modification, using a functional polycatecholamine layer, enables the immobilization of selected oxidoreductases (laccase and peroxidase) on curdlan hydrogel. The following significant changes of biological and mechanical properties of polycatecholamines + oxidoreductases-modified matrices were observed: reduced response of human monocytes in contact with the hydrogels, modulated reaction of human blood, in terms of hemolysis and clot formation, and changed mechanical properties. The lack of toxicity towards human fibroblasts and the suppression of cytokines released by human monocytes in comparison to pristine curdlan hydrogel, seems to make the application of such modifications attractive for biomedical purposes. The obtained results could also be useful for construction of a wide range of biomaterials based on other polymer hydrogels.

## 1. Introduction

Oxidoreductases, represented by laccase, are enzymes of wide interest in many fields, including that of medical applications. For example, fungi-isolated laccase were reported to exhibit anticancer and antitumor activity [1,2,3]. Laccase from *Cerrena unicolor* showed high prooxidative potential, which is likely to be a tool in their action towards pathogenic cells, such as *Escherichia coli* [4]. An example of laccase therapeutic activity is a treatment of poison ivy dermatitis caused by the plant toxin, urushiol. In oxidized form, urushiol is nontoxic and its oxidation can be performed by laccase [5]. Another potential application of this enzyme is oxidation of iodide to iodine, widely used as a disinfectant [5]. Moreover, it can catalyze the transformation of 4-methyl-3-hydroxyanthranilic acid to actinocin, which was found to be effective in the fight against cancer and the oxidative coupling of katarantine and vindoline to yield vinblastine, an important anti-cancer drug, especially useful in the treatment of leukemia [6]. Laccase from *Trametes versicolor* treatment together with the 2,2,6,6-tetramethyl-1-piperidinyloxy free radical increased the antioxidant activity of propolis and poplar bud exudates [7]. Laccase treatment was used for bioactive O-carboxymethyl chitosan (CMCS) hydrogel crosslinked with natural phenolics with potential application in wound dressings due to its antioxidant and anti-inflammatory properties [8]. Interestingly, pigments and other laccase products can reduce immune recognition, potentially interfering in signaling pathways of monocytes. Thus, laccase can mediate the activity of the immune system and play a role in the virulence factor of some opportunistic pathogens [9]. This feature of laccases may be used for modification of biomaterials which induce a high immune system response, leading to the modulation of the immune system and, potentially, to the discovery of novel functions of this enzyme [10]. Apart from laccases, other oxidoreductases may show potential in medical applications. For example, recombinant peroxidase coupled with indole-3-acetic acid formed a pro-drug system of extensive cytotoxicity against several cancer cell lines [11]. Horseradish peroxidase was also used for crosslinking of dopamine- and tyramine-conjugated poly(propylene oxide)-poly(ethylene oxide) to obtain adhesive and nontoxic hydrogels for biomedical applications [12]. Sialoperoxidase and myeloperoxidase found in the oral cavity can catalyze the oxidation of the pseudohalide anion thiocyanate (SCN-) to hypothiocyanite (OSCN-), which is a strong oxidant of antimicrobial role. This suggests possible applications of peroxidases in oral therapy [13].

For many reasons, related to bioremediation of various pollutants or medical applications, oxidoreductases have been immobilized on different hydrogels which provide appropriate and biocompatible environments for enzymatic reactions. For example, immobilized laccases from *T. versicolor* and *C. unicolor* were effectively applied for removal of persistent trace compounds in wastewaters (diclofenac, triclosan, paracetamol, bisphenol A, oils, dyes and other pollutants) [14,15,16,17,18,19]. Sampaio et al. [20] proposed the bacterial nanocellulose-based hydrogel with immobilized commercial laccase. This hydrogel exhibited antibacterial properties and cytotoxicity acceptable for wound dressings. Therefore, there is a reason to verify the potential of other oxidoreductases modified hydrogels for medical applications. One of the promising hydrogel matrices which can be considered for this application is curdlan (β-(1,3)-glucan) which forms low-set and high-set gels under thermal treatment. Recently, the following new possibilities of curdlan application in medicine emerged: (i) as solubilized and biologically active derivatives and (ii) as compounds of insoluble hydrogels designed for wound dressings, as an example [21,22,23,24,25]. As a biomaterial, curdlan shows not only benefits but also disadvantages. For example, in particulate form it may induce granuloma formation, microembolization and inflammation [26], although, according to some reports, high-set curdlan did not stimulate monocyte and macrophage response [27]. Another disadvantage of curdlan hydrogel is insusceptibility to immobilization of different molecules (including enzymes) due to its chemical structure and lack of highly reactive groups in glucose units. For this reason, curdlan requires pre-activation with active linkers before the molecules bind. A promising tool which can be used for this purpose is dopamine which was reported to form self-adhesive polydopamine (PDA) coatings on different organic and inorganic surfaces [28]. PDA coatings were used, for example, for the modification of graphene nanosheets [29,30], Fe_3_O_4_ nanoparticles [30,31,32], silica nanoparticles [33] or polymers [34,35,36]. Importantly, PDA coatings contain catechol domains which can react with thiols and amines via Michael addition or Schiff base reactions [37,38], thus allowing for secondary coupling reactions with different organic molecules. Polymerized L-DOPA (PLD) and norepinephrine (PNE) were recently proved to act similarly to PDA, namely, they can adhere to solid matrices (polysaccharide hydrogel, polyester fibers, cellulose membranes) [39,40,41]. Both dopamine and L-DOPA polymerize in the presence of oxidants (as O_2_ or Cu^2+^ ions) and in slightly alkaline buffers. In our recent works, we deposited the PDA and PLD coatings on high-set curdlan hydrogel [36,39]. Both types of the hydrogel revealed an increased soaking capacity and stability in human blood and serum, accelerated blood clot formation, reduced the tendency of fibroblast adhesion and revealed nontoxicity in the Danio rerio model. Both PDA- and PLD-coated curdlan hydrogels were coupled with gentamicin, resulting in modified curdlan hydrogels of high antibacterial activity [36,39]. The obtained results suggest that PDA- and PLD-coated curdlan hydrogels show high potential in the treatment of infected wounds and other biomedical applications.

Therefore, we prepared PDA- and PLD-coated curdlan hydrogels. Then we used PDA and PLD coatings as platforms (linkers) for laccase and peroxidase immobilization via free amino groups in protein molecules and catechol domains in polycatecholamines. According to our hypothesis, these enzymes may decrease the immune stimulatory activity exhibited by curdlan via modulation of inflammatory mediators synthesis [42]. This could increase the biomedical potential of curdlan hydrogels because stimulation of immune response by biomaterials is likely to cause side effects and to prolong wound healing time. The following other potential features of the modified curdlan hydrogels were also evaluated with respect to their potential use as wound healing biomaterials: interactions with human blood and resistance to mechanical compression. This article presents the obtained results and perspectives of their impact on potential applications in medicine.

## 2. Results and Discussion

### 2.1. Laccase and Peroxidase Immobilization

Complete lack of laccase and peroxidase presence was shown for pristine (uncoated) curdlan hydrogel (both in terms of protein amount and enzymatic activity) (Table 1). This observation is logical because curdlan hydrogel does not possess sufficiently active chemical groups in glycosyl units of triple helices, capable of binding to other molecules (only weakly active hydroxyl groups are present in curdlan’s structure). This suggested that the linker between the hydrogel network and protein molecules was necessary for enzyme immobilization. For this purpose, mild modification of curdlan by deposition of polycatecholamine on the hydrogel was proposed.

Modification of curdlan hydrogel samples with polydopamine (PDA) or poly (L-DOPA) (PLD), both with and without the addition of copper ions to the reaction mixture, resulted in the formation of uniformly black slices compared to white control slices, as described earlier [36]. The black color indicated the formation of polycatecholamine coating on the curdlan hydrogel. After the coating, the hydrogel slices were carefully washed to remove all reagents, including copper ions and non-adhered PDA and PLD, to avoid undesired immobilization of enzymes to free polycatecholamines. Copper ions were earlier reported to act as an oxidant for dopamine polymerization, resulting in the formation of thicker polydopamine layers in comparison with the same deposition process using oxygen as the oxidant [43]. Most probably, PLD thickness is the main factor responsible for this phenomenon. According to the XPS result of the pilot experiment, the content of copper in the prepared samples was too low (0.05 %At Conc., data not shown) to suspect that it was incorporated into the coating and could play a significant role in protein binding. The amount of immobilized laccase or peroxidase on such modified curdlan hydrogels varied within the range 1625–2755 µg protein/g dry hydrogel weight or 2386–3608 µg protein/g dry hydrogel weight, respectively (Table 1). According to the results, the coatings deposited in assistance of copper ions were capable of immobilizing both enzymes with a higher immobilization yield, which is in agreement with our earlier results for gentamicin immobilization of PLD-coated polyester vascular prostheses [40]. Moreover, the hydrogel coating using PLD was more beneficial in comparison with PDA, for both enzymes (Table 1). Therefore, for further experiments, PLD-coated hydrogels were selected and immobilized enzyme activity was measured for these samples. For enzymatic activity, especially in the case of laccase, the hydrogel coating with PLD in assistance of copper ions resulted in higher activity of immobilized enzymes.

**Figure 1:**
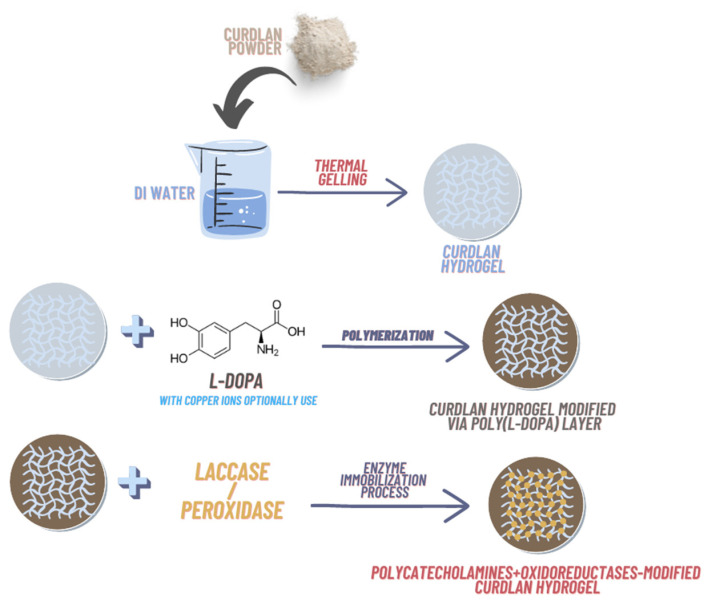
Scheme of hydrogels synthesis.

Designation of hydrogel samples selected for further testing and their images were presented in Table 2 and the synthesis scheme in Figure 1.

### 2.2. Cytotoxicity Tests

**Figure 2:**
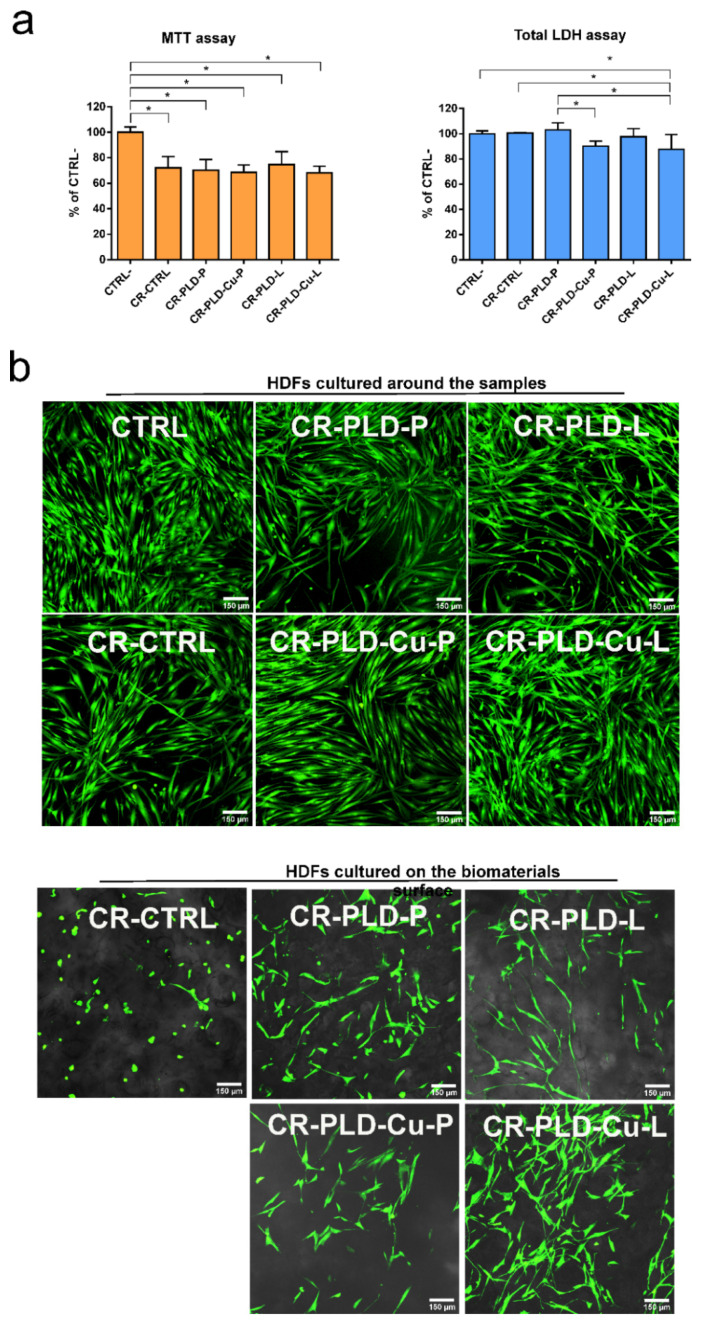
Cytotoxicity tests against human dermal fibroblasts (HDFs): (**a**) quantitative evaluation of cytotoxicity by MTT test (cell metabolism) and total LDH assay (cell number); CTRL−-negative control of cytotoxicity revealed 100% viability; * statistically significant (*p* < 0.05) results between indicated group according to One-way ANOVA with post-hoc Tukey test; (**b**) qualitative evaluation of cytotoxicity by live/dead staining (green fluorescence-viable cells, red fluorescence-dead cells) followed by CLSM observation; CTRL-control cells grew on polystyrene well.

Cytotoxicity of the biomaterials was assessed quantitatively by measurement of two parameters: cell metabolism (MTT assay) and cell number (total LDH test) (Figure 2a). MTT assay clearly showed that all tested samples, including unmodified control material (CR-CTRL), significantly reduced cell metabolism to approximately 60–70% compared to the negative control (CTRL– cells revealing 100% viability). Interestingly, evaluation of cytotoxicity by total LDH assay proved that all biomaterials were nontoxic, as cell viability (expressed as cell number in population) was above 70% compared to CTRL–. According to ISO 10993-5, an extract of the biomaterial is nontoxic when cell viability is higher than 70%. The extracts of the samples produced with the use of Cu^2+^ ions (as additional oxidant) showed the trend to decrease cell viability in comparison with the samples produced without copper ions (to approximately 85–90% compared to control cells (CTRL–)). However, only for CR-PLD-Cu-P in comparison with CR-PLD-P was this difference significant.

The explanation of this trend could be related to the presence of Cu^+2^ ions in the samples because Cu^2+^ ions reveal dose-dependent cytotoxicity against eukaryotic cells [44]. However, according to XPS data, the Cu ions present in PLD-coated hydrogels was insignificant and doubtful. Another explanation of this phenomenon is the cytotoxic activity of laccase, which is known to exert a negative effect on the viability of fibroblasts [45,46]. Hydrogels coated with the use of Cu^2+^ ions were shown to bind more enzymatic protein and revealed higher enzymatic activity (Table 1). Thus, these samples were also likely to cause an increased cytotoxicity in comparison with samples containing less enzyme.

Nontoxicity of the tested samples was confirmed by qualitative experiment performed in direct contact with human fibroblasts. Live/dead staining clearly showed a monolayer of viable cells (green fluorescence) with unchanged morphology growing on the polystyrene surface around the tested biomaterials (Figure 2b). All modified samples were also supportive to fibroblast adhesion as CLSM images showed well spread viable fibroblasts with typical lengthened morphology on their surfaces (Nomarski contrast was used to show microstructure of the samples). However, unmodified control biomaterial (CR-CTRL), despite nontoxicity, was unfavorable to cell adhesion. Fibroblasts cultured on this sample were viable (green fluorescence) but had a spherical shape. Only single dead cells (red fluorescence) were detected in direct contact experiment, proving the nontoxic character of the samples. These results were in agreement with the findings of other groups. For example, Oh et al. prepared adhesive hydrogel by horseradish peroxidase-mediated crosslinking and found it to be nontoxic for human fibroblasts [12].

**Figure 3:**
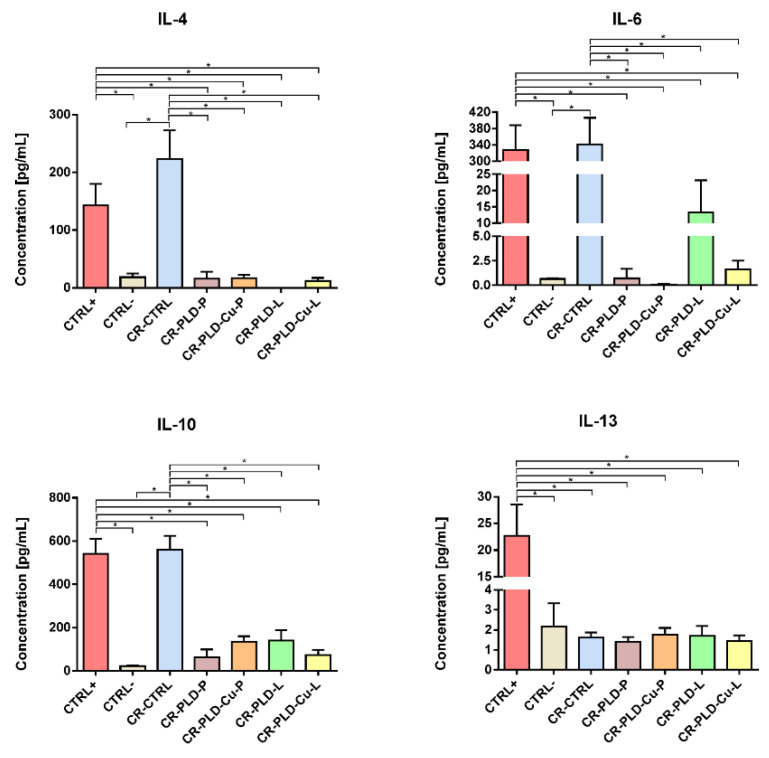
Cytokine release by human monocytes (THP-1 cells) assessed by ELISAs; CTRL+-positive control in the form of cells stimulated with pro-inflammatory factors (LPS, INF-γ), CTRL−-negative control in the form of untreated cells cultured on polystyrene well; * statistically significant (*p* < 0.05) results between indicated group according to One-way ANOVA with post-hoc Tukey test.

In the study of cytokine release by monocytes, the release profiles of pro-inflammatory IL-6 and three anti-inflammatory cytokines (IL-4, IL-10, and IL-13) were determined. In the case of samples marked as CR-PLD-P, CR-PLD-Cu-L, and CR-PLD-Cu-P, the release profiles of IL-4 and IL-6 were comparable to the negative control (CTRL−-untreated cells) (Figure 3). Interestingly, CR-PLD-L biomaterial reduced IL-4 release to an undetectable level by ELISA and significantly increased IL-6 production compared to CTRL–. Thus, it may be assumed that a reduced level of anti-inflammatory cytokine (IL-4) in response to the CR-PLD-L sample resulted in increased synthesis of pro-inflammatory cytokine (IL-6). The release of IL-10 by monocytes cultured in the presence of all modified samples was slightly elevated (but without statistical significance) compared to CTRL–; however, the IL-10 level did not reach that of the positive control (CTRL+-cells stimulated with LPS and INF-γ). The unmodified control sample (CR-CTRL) significantly increased production of IL-4 and IL-6, IL-10 by monocytes to levels similar to CTRL+. Analysis of IL-13 release did not reveal any statistically significant differences between all tested samples, including CR-CTRL. Monocytes cultured in the presence of all biomaterials produced comparable levels of IL-13 to CTRL–, and significantly reduced amounts of IL-13 compared to CTRL+. It is not surprising that the CR-CTRL sample promoted cytokine release by monocytes since curdlan and other β-glucans are proven to be immunomodulatory factors, having the ability to activate immune cells [47,48,49]. Considering the observed cytokine release profile, it might be inferred that implantation of an unmodified CR-CTRL sample carried high risk of biomaterial-induced immune response since cells cultured in the presence of the biomaterial revealed comparable cytokine profile release to monocytes stimulated with LPS and INF-γ (CTRL+), whereas applied modifications of the CR-CTRL sample significantly reduced its immunomodulatory properties, making it more safe for biomedical applications.

### 2.3. Interactions with Human Blood

**Figure 4:**
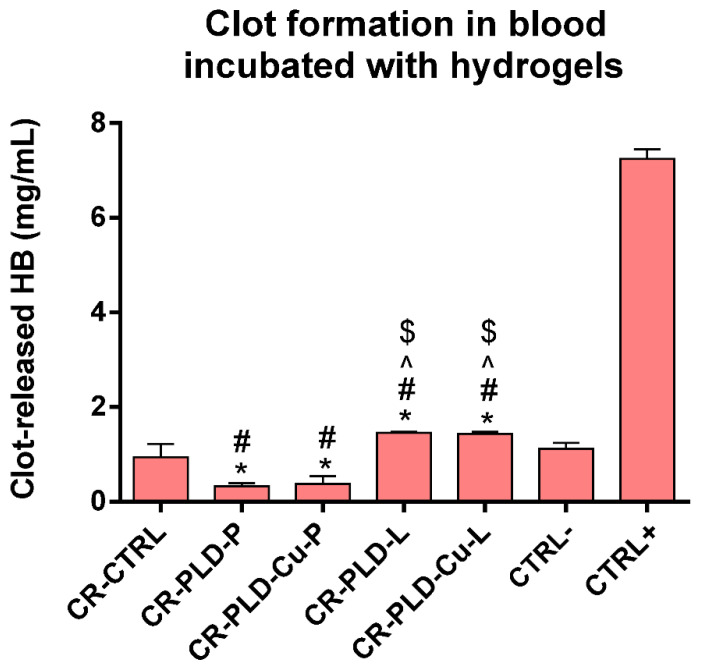
Clot formation in blood incubated with control and poly(L-DOPA)-modified curdlan hydrogel samples with immobilized oxidoreductases. (*) symbol indicates statistically significant differences between the negative control and other samples, (#) symbol indicates statistically significant results between CR-CTRL sample and other samples, (^) symbol indicates statistically significant results between CR-PLD-P sample and the other samples, ($) symbol indicates statistically significant results between CR-PLD-Cu-P sample and other samples. *, #, ^, $ symbols indicate statistically significant results according to One-way ANOVA with post-hoc Tukey’s test for 30 min (*p* < 0.05).

Interactions with blood are an important aspect of wound dressing–human blood interactions, especially for bleeding wounds. For this reason, the clot forming ability of the blood was verified during 30 min of contact with the samples. In comparison with CTRL− (negative control), CR-CTRL reference hydrogel resulted in similar clot-forming capacity (Figure 4). The pro-clotting effect was distinct for both peroxidase-bound coatings, with significant difference in comparison with negative control and uncoated curdlan hydrogel. On the other hand, a slight but significantly different anti-clotting effect was observed for both laccase-bound coatings, in comparison with negative control and uncoated curdlan hydrogel (Figure 4). This suggested that modification of curdlan hydrogel with PLD + laccase complex was almost neutral for blood clotting capacity, while PLD + peroxidase coating enhanced the clot-forming ability. Use of copper ions for PLD-mediated oxidoreductase immobilization did not affect the clotting ability of blood incubated with the studied hydrogels.

**Figure 5:**
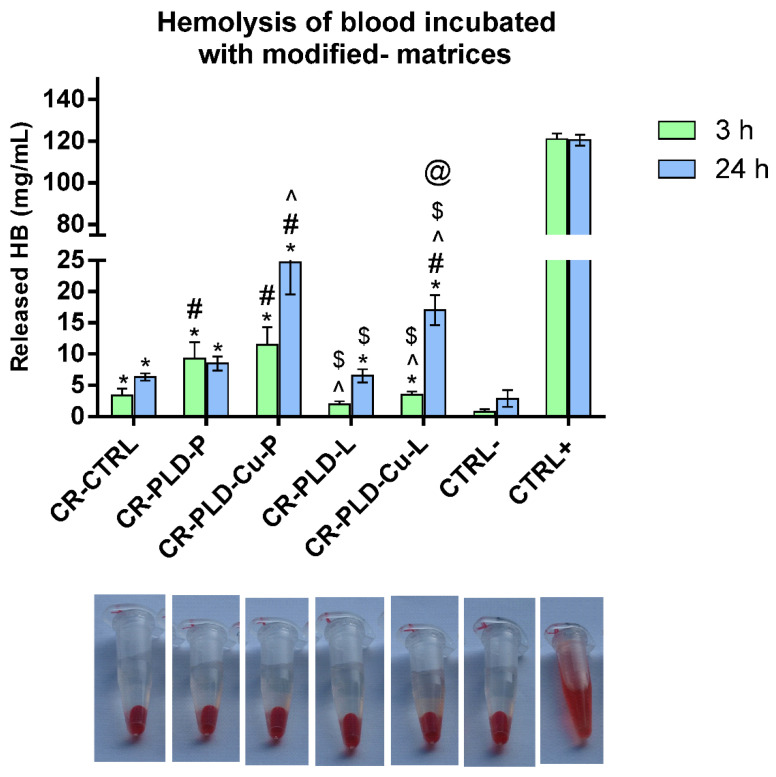
Hemolysis in blood incubated with control and poly(L-DOPA)-modified curdlan hydrogel samples with immobilized oxidoreductases. (*) symbol indicates statistically significant differences between the negative control and other samples, (#) symbol indicates statistically significant results between CR-CTRL sample and other samples, (^) symbol indicates statistically significant results between CR-PLD-P sample and other samples, ($) symbol indicates statistically significant results between CR-PLD-Cu-P sample and other samples, (@) symbol indicates statistically significant results between CR-PLD-L sample and other samples. *, #, ^, $, @ symbols indicate statistically significant results according to One-way ANOVA with post-hoc Tukey’s test for 3 h and 24 h (*p* < 0.05), respectively in comparison with appropriate sample variants for each time point.

Results of the test of blood hemolysis led to different conclusions. First, uncoated curdlan (CR-CRTL) hydrogel caused statistically significant hemolysis, both after 3 h and 24 h of contact (Figure 5). Modification of the hydrogel with PLD-oxidoreductase complexes notably increased the blood hemolysis in comparison with negative control. Only CR-PLD-L hydrogel did not cause blood hemolysis after 3 h (Figure 5). Further analysis of the results, in particular for 24 h, also showed that hydrogels coated with PLD + oxidoreductases in the presence of Cu ions significantly enhanced blood hemolysis in comparison with pure curdlan (CR-CTRL). PLD-coatings on curdlan hydrogel were earlier reported to only minimally increase the blood hemolysis [39]. However, in the cases of CR-PLD-Cu-L and CR-PLD-Cu-P hydrogels, the hemolysis increased to approximately 15–20% of the positive control (Figure 5). Thus, the observed pro-hemolytic effect of modified hydrogels appeared and was probably due to the higher amount of immobilized laccase and peroxidase (Table 1). For complexes deposited without Cu ions (CR-PLD-L and CR-PLD-P), and with lower amounts of enzymatic proteins, their hemolytic activity was not significant (Figure 5). Moreover, the presence of peroxidase caused higher hemolysis than laccase in most cases, and this effect was statistically different (Figure 5).

Explanation of this phenomenon may lie in oxidoreductases properties. Unfavorable excessive peroxidase activity is implicated in oxidative damage of cells and tissues. Generation of, for example, superoxide radicals may induce the redox activity of hemoproteins, causing damage in plasma proteins as well as lipid peroxidation and fragmentation that may result in erythrocyte membrane damage [50]. It was also reported that peroxidase (and several additional proteins) present in eosinophil granules, can stimulate platelet activation and aggregation [51]. In the case of C. unicolor laccase, its prooxidative potential was previously reported by Jaszek at al. [4]. Additionally, the presence of Cu^2+^ ions during the coating process may induce a slight hemolytic effect [52]. This last explanation is unlikely because the hydrogels were carefully washed before the tests and the copper content in the samples, according to XPS, was extremely low (on the edge of XPS detection limit). It should be noted, however, that hydrogels produced without copper ions addition were relatively safe in contact with blood (Figure 5). Therefore, the results suggest the potential of the presented modification method in medical applications.

### 2.4. Mechanical Properties

**Figure 6:**
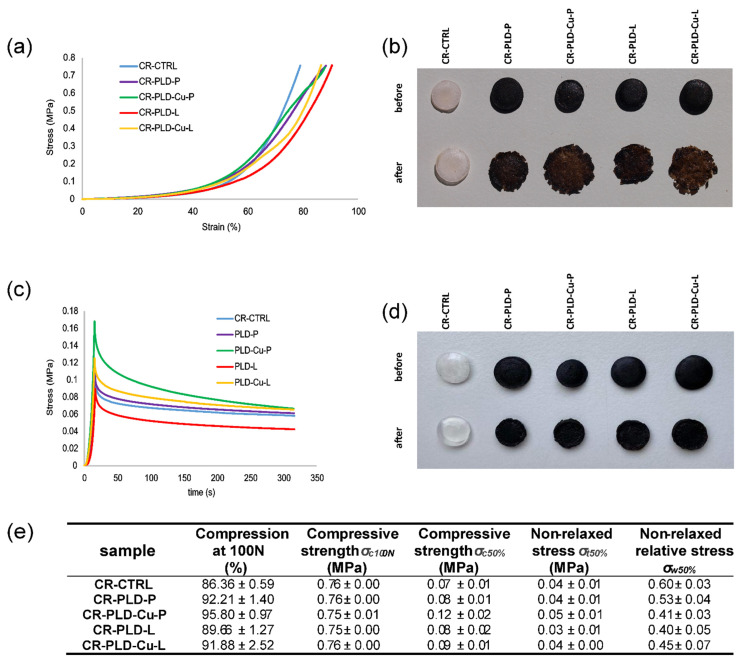
Mechanical properties of control and PLD + oxidoreductases-modified hydrogels. (**a**) representative stress–strain curves for compression to 100N; (**b**) images of the specimens before and after compression to 100N; (**c**) representative stress–strain curves for compression to 50% and relaxation; (**d**) images of the specimens before and after relaxation; (**e**) mechanical parameters of the hydrogels.

Resistance of wound dressing to mechanical pressure is important in view of its practical use. Representative stress–strain curves, as well as the compression–relaxation curves are presented in Figure 6a,c. The stress–strain curves obtained for all PLD + oxidoreductases-modified curdlan hydrogels, as well as for pristine curdlan hydrogel, were subjected to compression with a force of 100N, and showed a nonlinear increase in compressive stress in relation to strain (Figure 6a). Compressive strength was also similar for all samples (Figure 6e). The curves showed similarity to elastomers’ behavior and indicated the viscoelastic behavior of the tested hydrogels. However, control hydrogel resisted the pressure of 100N without a notable structural change while all PLD + oxidoreductases-modified hydrogels were significantly damaged (Figure 6b). Moreover, samples containing higher amounts and activities of enzymes (CR-PLD-Cu-L and CR-PLD-Cu-P) were more disrupted than samples containing less enzymes (CR-PLD-L and CR-PLD-P; Figure 6b). This visual observation was in agreement with values of hydrogels compression at 100N: approximately 86% for pristine curdlan and approximately 89–96% for modified curdlan (Figure 6e). It seems that PLD + oxidoreductases modification of curdlan reduced its elasticity, with simultaneous increase of plasticity. The shape of the curves suggested the lack of distinct damages in the structure of the samples which may be related to the homogenous impairment of the stability of curdlan helices (Figure 6a).

The samples compressed to only 50% of strain and left for relaxation did not show significant structural damage, as shown in Figure 6d. The shapes of all curves were similar. However, the obtained values of non-relaxed relative stress (*σ_w_*) pointed at dominance of elastic behavior for the control (CR-CTRL) sample, while PLD + oxidoreductases-modified hydrogels were found to be more viscous (Figure 6e). These results are in agreement with results of compression with a force of 100 N (Figure 6e). Therefore, the results of the relaxation test confirmed the observations made during the compression with a force of 100 N.

The question is, why did the presence of oxidoreductases cause the shift of the hydrogel behavior from elasticity to viscosity. The polycatecholamine is unlikely to cause this effect because polydopamine was already shown to exert no destructive impact on curdlan hydrogel’s mechanical properties [36]. More possibly, the polydopamine complex underwent degradation by the free radicals produced by both enzymes which, in turn, resulted in the reduced stability of the curdlan hydrogel structure. In the case of laccase, polydopamine may act as a form of mediator transferring an unpaired electron to other compounds that are not natural substrates for this enzyme, including curdlan [53,54,55].

In summary, some statements may be made in this pilot study. First, laccase and peroxidase cannot be immobilized directly on pristine curdlan hydrogel and a linker between protein molecules and curdlan fibers is absolutely required. Second, polycatecholamines can be stably deposited on curdlan hydrogel and allow for immobilization of laccase and peroxidase. Third, the presence of PLD-oxidoreductases modification in curdlan hydrogels changes the properties of pristine curdlan hydrogel. Regarding the latter, the following changes in properties are relevant: the presence of PLD-oxidoreductases alters the response of human monocytes in contact with the hydrogels, affects the reaction of human blood in terms of hemolysis and clot formation and changes the mechanical properties of hydrogels. Fourth, although the same PLD coatings were deposited in all tested samples, the properties of the modified hydrogels were altered to different extents. For example, the level of cytokines release by monocytes was hardly affected, despite the used modification. The blood clot formation was similar for peroxidase and laccase but was changed depending on the enzyme amount and activity. Blood hemolysis and mechanical properties of matrices seemed to be changed by the amount of immobilized enzyme, rather than its type.

These interesting observations may serve as a starting point for further studies on the medical applications of oxidoreductases in the design of hydrogels-based biomaterials, including curdlan. It should be highlighted that applied modification of curdlan hydrogel reduces the risk of biomaterial-induced immune response, making it safer for biomedical applications. It is especially interesting in light of the fact that curdlan is common, cheap, and biodegradable but is still an underestimated biopolymer. The introduced PLD-oxidoreductases modification seems to increase the potential of its biomedical applications. The observed disadvantages are likely to be limited by decrease of immobilized enzymes or introduction of antioxidants to diminish the prooxidative potential of enzymes. The change of the linker between oxidoreductases and the curdlan matrix is another possibility, which could be useful for the improvement of oxidoreductases-modified hydrogels.

## 3. Materials and Methods

### 3.1. Laccase Production and Purification

Laccase was purified from *Cerrena unicolor* (Bull. Ex Fr.) Murr, strain: FCL139 (Fungal Collection of Lublin at the Department of Biochemistry and Biotechnology, Maria Curie-Skłodowska University, ITS sequence GenBank accession number: DQ056858) post-culture fluid. The mycelium was grown in 10 L airlift bioreactors containing 8 l of Lindeberg-Holm’s medium, with aeration 15 L/min. After 10 days, the culture fluid was separated from the mycelium by filtration and centrifugation, and, then, it was subjected to 10 kDa cut-off, membrane ultrafiltration. After freeze-drying (Labconco, Kansas City, MO, USA), the preparation was subjected to gel-filtration chromatography on a Sephadex G-50 (5.0 × 20 cm bed dimensions), followed by DEAE Sepharose anion exchange chromatography The 2.5 × 15 cm column was equilibrated with 20 mM Tris-HCl buffer (pH 6.5). Elution was conducted with a linear NaCl gradient (0.1–0.5 M) at a 1 mL/min flow rate for 360 min. Fractions containing laccase activity were pooled, desalted on a Sephadex G-50 column and lyophilized [4].

### 3.2. Synthesis of Polycatecholamine-Coated Curdlan Hydrogels

Curdlan powder (from *Alcaligenes faecalis*; cat. No. 281-80531; DP 6790; specific rotation [A]20/D: +30~+35) was purchased by Wako Chemicals (Japan); Tris (2-Amino-2-(hydroxymethyl)propane-1,3-diol), dopamine and L-DOPA (3,4-Dihydroxy-L-phenylalanine) by Sigma-Aldrich (Burlington, MA, USA). Control and polycatecholamine-coated hydrogels were synthesized according to the method described elsewhere (Michalicha et al., 2021). Briefly: a suspension of 0.8 g curdlan in 10 mL 10 mM Tris/HCl buffer pH 8.5 was polymerized in glass tubes (ø 13 mm) at 93 °C for 15 min. After cooling, the hydrogel was cut into 3 mm slices. Some slices were used without further modification as controls. Other slices were incubated in 5 mL 10 mM Tris/HCl buffer pH 8.5 containing 10 mg of dopamine or L-DOPA, in an orbital shaker for 24 h at 25 °C, to allow L-DOPA polymerization. In some versions, the mixture was supplemented with 0.5 mM CuSO_4_ (oxidant alternative to O_2_). Afterwards, the coated slices were washed approximately 10 times in 100 mL DI H_2_O, to remove all non-reacted compounds and non-adhered polycatecholamine, frozen at −20 °C and lyophilized.

### 3.3. Enzymes Immobilization and Quantitative Analysis

Immobilization of laccase and peroxidase on uncoated and polycatecholamine-coated curdlan hydrogels was performed by incubation of lyophilized slices (ø 13 mm. 3 mm) in 0.5 mg/mL laccase or peroxidase (Sigma-Aldrich, USA) in Britton-Robinson buffer pH 8.5, in proportion 33.3 mL of enzyme solution/1 g lyophilized curdlan hydrogel slices. The process included 12 h of shaking at 25 °C (DTS-4 shaker, 100 rpm), followed by 36 h incubation at 4 °C for laccase and 24 h incubation at 4 °C for peroxidase. Then, slices were washed in 10 mL DI water, frozen at −20 °C and lyophilized.

The amount of immobilized enzyme was measured quantitatively from the difference between protein concentration in laccase or the peroxidase solution before and after incubation with curdlan hydrogels. Protein concentration was estimated according to Schacterle-Pollack method [56]. The results were presented in µg protein/g of dry hydrogel weight.

### 3.4. Laccase and Horseradish Peroxidase Assay

Immobilized laccase activity of 15 mg curdlan hydrogels was measured spectrophotometrically in a stirred cuvette with 0.025 mM of syringaldazine (4-hydroxy 3,5-dimetoxybenzaldehyde) in 50 mM citrate-phosphate buffer at pH 5.3 (2 mL final volume). The oxidation of the substrate was recorded at 525 nm at 30 °C for 60 s. One unit of activity was defined as the amount of the enzyme required to oxidize 1 mole of syringaldazine per sec and expressed in units (U) per 1 g of dry hydrogel [4,57].

Peroxidase activity of 15 mg curdlan hydrogels was measured against ABTS (2,2′-azino-bis(3- ethylbenzthiazoline-6-sulfonic acid)) and hydrogen peroxide solution. An amount of 3.0 mL (end volume) of the reaction mixture with a final concentration of 96 mM of potassium phosphate buffer, pH 5, 8.7 mM ABTS and 0.01% (*v/v*) hydrogen peroxide was added to the stirred cuvette containing hydrogels. The absorbance was measured continuously over the period of 3 min at 405 nm. One unit of enzyme activity oxidized 1.0 µmole of ABTS per minute at pH 5.0 at 25 °C. The results were expressed in units per 1 g of dry hydrogel [58,59].

### 3.5. Cell Culture Tests

Cell culture tests were carried out with the use of primary human dermal fibroblasts (HDFs, ATCC-LGC Standards) and human acute monocytic leukemia cells (THP-1 cell line, ATCC-LGC Standards). The HDFs and THP-1 cells were maintained at 37 °C (humidified atmosphere of 5% CO_2_ and 95% air) in culture media recommended by ATCC-LGC Standards; Fibroblast Basal Medium was supplemented with the components of Fibroblast Growth Kit-Low Serum (both purchased from ATCC-LGC Standards) and RPMI medium (ATCC-LGC Standards) was supplemented with 0.05 mM 2-mercaptoethanol (Sigma-Aldrich Chemicals) and 10% fetal bovine serum (FBS, Pan-Biotech GmbH), respectively.

### 3.6. Cytotoxicity Tests on Human Dermal Fibroblasts

Cytotoxicity of the biomaterials was assessed according to ISO 10993-5 standard using fluid extracts prepared according to ISO 10993-12 standard, as described previously (Michalicha, 2021) [39]. Suspension of HDFs was seeded at a density of 2 × 10 ^4^ cells/well into the wells of 96-multiwell plates. The cells were cultured at 37 °C for 24 h and then the culture medium was discarded and fibroblasts were exposed to the extracts of the samples. Control cells were exposed to the complete culture medium treated in the same manner as biomaterials during the extraction procedure. The cytotoxicity of the samples was assessed after 24 h culture of the fibroblasts in the extracts. Viability of cells was determined based on their metabolic activity by MTT assay (Sigma-Aldrich Chemicals) and lactate dehydrogenase (LDH) release after cell lysis (total cell number) by total LDH test (In Vitro Toxicology Assay Kit-TOX7, Sigma-Aldrich Chemicals). The MTT assay was conducted using the procedure described previously (Przekora, 2014) [60]. The total LDH test was carried out according to the manufacturer’s instructions. The cytotoxicity results were presented as the percentage of control cells (negative control of cytotoxicity-CTRL−) that were supposed to show 100% viability. Both assays were conducted in quadruplicate in three independent experiments. Statistically significant differences between the samples were considered at *p* < 0.05 according to One-way ANOVA with post-hoc Tukey test (GraphPad Prism 8.0.0 Software, San Diego, CA, USA).

Cytotoxicity of biomaterials was also evaluated by direct contact with the fibroblasts using Live/Dead Double Staining Kit (Sigma-Aldrich Chemicals) and confocal laser scanning microscope (CLSM) with Nomarsky contrast observation. Prior to the test, the samples (discs having 12 mm in diameter and 2 mm in height, weighing 30 mg ± 2 mg) were put into the wells of 24-multiwell plate and preincubated in the complete culture medium. HDFs were seeded directly on the biomaterials at a density of 2 × 10^5^ cells/sample. Cells grown on a polystyrene well of the 24-multiwell plate served as control cells (CTRL), showing high viability and good adhesion. Then, 72 h after seeding, HDFs were stained according to the manufacturer protocol using green fluorescent calcein-AM (viable cells) and red fluorescent propidium iodide (dead cells). Stained HDFs were analyzed under CLSM (Olympus Fluoview equipped with FV1000).

### 3.7. Cytokine Release by Monocytes

Based on all tests, 4 samples were selected for further cell culture characterization: CR-PLD-L, CR-PLD-P, CR-PLD-Cu-L, and CR-PLD-Cu-P. The samples were put into the wells of 24-multiwell plate and preincubated in the complete culture medium. Human THP-1 monocytes were seeded directly on the biomaterials at the density of 2.5 × 10^5^ cells/sample. Monocytes grown in a polystyrene well of the 24-multiwell plate without biomaterial served as negative control (CTRL−), showing a physiological level of cytokine release. Monocytes cultured in a polystyrene well of the 24-multiwell plate in the medium supplemented with 100 ng/mL of lipopolysaccharide (LPS) and 25 ng/mL of interferon-gamma (INF-γ, Sigma-Aldrich Chemicals) served as positive control (CTRL+), showing increased level of cytokine release due to stimulation with pro-inflammatory factors. After 48-h culture, cell culture supernatants were harvested to assess concentrations of interleukin (IL)-4, IL-6, Il-10, and IL-13 by human specific ELISA kits (EIAab). ELISAs were carried out according to the manufacturer’s protocol using three independent samples. Statistically significant differences between the samples were considered at *p* < 0.05 according to#]p098 One-way ANOVA with post-hoc Tukey test (GraphPad Prism 8.0.0 Software, San Diego, CA, USA).

### 3.8. Blood Compatibility Tests

Hemolysis and blood clot formation was performed on coated and uncoated (control) hydrogels slices (in triplicate), as described elsewhere [23]. Briefly, human citrated blood for the tests was collected from a healthy volunteer, according to a procedure approved by the Bioethics Committee at the Medical University of Lublin (agreement no KE-0254/258/2020). Its total hemoglobin and plasma hemoglobin concentration was measured on the basis of the reaction with Drabkin reagent (Chempur, Poland) and calibration curve on human hemoglobin (Sigma-Aldrich, USA), using 96-well plates and Synergy H4 hybrid microplate reader, Biotek, USA). Total hemoglobin and plasma hemoglobin concentration in the collected blood were 140 mg% and 0.13 mg/mL, respectively. In both tests, pieces of HDPE (high density polyethylene, Sigma-Aldrich, USA), of approximately 130 mm^2^ surface, were used as negative control (CTRL−; the surface corresponding to the surface of curdlan hydrogel slices). Positive control (CTRL+) in the hemolysis test contained 0.1% Triton X-100 test while in the blood clot formation test it was represented by non-activated Ca^2+^-free whole blood.

Hemolysis was evaluated as a function of hemoglobin released from erythrocytes destroyed during 3 h and 24 h contact between the hydrogel slices and blood diluted 100-fold in PBS pH 7.4 (5 mL/50 mg of the hydrogel) at 37 °C. Clot formation was estimated by incubation of the samples with 100 µL whole CaCl_2_-activated blood for 30 min at 37 °C, followed by measurement of hemoglobin in free erythrocytes. This measurement was based on the inverse relationship between the concentration of hemoglobin released from free erythrocytes and the efficiency of blood clot formation. The hemoglobin concentration in both measurements was evaluated using Drabkin reagent, as described above. Statistically significant differences between the samples were considered at *p* < 0.05 according to One-way ANOVA with post-hoc Tukey test (GraphPad Prism 8.0.0 Software, San Diego, CA, USA).

### 3.9. Mechanical Parameters Evaluation

The mechanical characteristics of the tested materials were assessed using the EZ Test EX-SX universal testing machine (Shimadzu, Kyoto, Japan) equipped with the Trapezium program and a force sensor of 100 N. The compression test was carried out for hydrogel samples (ø = 13 mm; *n* = 10) completely soaked in PBS pH 7.4 with a crosshead rate of 5 mm/min. Measurements started after obtaining a force value of 0.05 N to eliminate gaps between the sample and the grips. The mechanical compression was carried out until 100 N force was reached. The obtained results determined compressive strength (*σ_c100N_*), measured as the maximum stress at 100 N.

The stress relaxation test was carried out by compression of the samples (ø = 13 mm; *n* = 10) until 50% of strain was reached. Then, the crosshead was stopped and the force was measured for 5 min. The relaxation test allowed for the determination of compressive strength (*σ_c50%_*), non-relaxed stress *σ_t50%_* and non-relaxed relative stress *σ_w50%_* calculated as the ratio *σ_t50%_/σ_c50%_*.

## 4. Conclusions

According to our observations, PLD-oxidoreductase-modified curdlan hydrogel showed wide possibilities in practical biomedical use. However, further studies in order to solve some technical problems are required. On the one hand, the modified hydrogels were found to be non-toxic for fibroblasts, and they also attenuated the cytokine release by human monocytes under contact with the hydrogel. These findings suggest that the proposed modification may increase the biological safety of curdlan hydrogel for biomedical applications as it reduces the risk of its rejection as a foreign body by the patient’s organism. On the other hand, the presence of PLD-oxidoreductases coatings slightly increased blood hemolysis and decreased the mechanical resistance of the modified curdlan hydrogels. These features of biomaterials designed as wound dressings seem to be unfavorable. However, these undesired aspects can be reduced, as proposed above.

The obtained results provided new data concerning the biomedical application of oxidoreductases, showing some critical points of the presented experimental model. This is especially interesting in relation to the design of other polymer-based hydrogels which could be useful for construction of a wide range of biomaterials. Therefore, the presented concept deserves further studies.

## Figures and Tables

**Table 1:** Results of laccase and peroxidase immobilization on polycatecholamine-coated curdlan hydrogel.

Amount of Immobilized Enzyme (µg/g Dry Hydrogel)	Type of Coating on Curdlan Hydrogel				
	Polydopamine (PDA)		Poly(L-DOPA) (PLD)		None
	–	+Cu^+2^	–	+Cu^+2^	
laccase	1625	1815	2520	2755	0
peroxidase	2386	3072	3430	3608	0
Activity of immobilized enzyme (U/g dry hydrogel)			poly(L-DOPA) (PLD)		none
			–	+Cu^+2^	
laccase			9.375	57.437	0
peroxidase			5.312	12.812	0

**Table 2:** Sample codes and images.

Sample Code	Sample Description	Sample Image
CR-CTRL	curdlan hydrogel	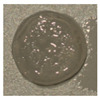
CR-PLD-P	curdlan hydrogel coated with poly(L-DOPA) + peroxidase	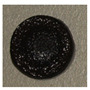
CR-PLD-Cu-P	curdlan hydrogel coated with poly(L-DOPA) with Cu^+2^ ions + peroxidase	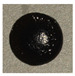
CR-PLD-L	curdlan hydrogel coated with poly(L-DOPA) + laccase	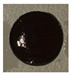
CR-PLD-Cu-L	curdlan hydrogel coated with poly(L-DOPA) with Cu^+2^ ions + laccase	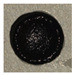

## Data Availability

Not applicable.
